# Prevalence and risk factors of abnormal left ventricular geometrical patterns in untreated hypertensive patients

**DOI:** 10.1186/1471-2261-14-136

**Published:** 2014-10-04

**Authors:** Hui Li, Fei Pei, Liying Shao, Jingzhou Chen, Kai Sun, Xinyu Zhang, Channa Zhang, Jibing Liu, Chuanshi Xiao, Rutai Hui

**Affiliations:** Department of Cardiology, The First Hospital Affiliated to Shanxi Medical University, 85 Jiefang South Road, Taiyuan City, Shanxi Province 030001 China; Sino-German Laboratory for Molecular Medicine, State Key Laboratory of Cardiovascular Disease, Fuwai Hospital, National Center for Cardiovascular Diseases, Chinese Academy of Medical Sciences and Peking Union Medical College, 167 Beilishilu, Beijing, 100037 China; Department of Cardiology, Bethune First Hospital of Jilin University, 71 Xinmin Street, Changchun City, Jilin Province 130021 PR China

**Keywords:** Left ventricular hypertrophy, Left ventricular geometry, Risk factors, Untreated hypertension

## Abstract

**Background:**

The various prevalence of LVH and abnormal LV geometry have been reported in different populations. So far, only a few reports are available on the prevalence of LV geometric patterns in a large Chinese untreated hypertensive population.

**Methods:**

A total of 9,286 subjects (5167 men and 4119 women) completed the survey and 1641 untreated hypertensive patients (1044 males and 597 females) enrolled in the present study. The LV geometry was classified into four patterns: normal; abnormal,defined as concentric remodeling;concentric or eccentric hypertrophy based on the values of left ventricular mass index (LVMI) and relative wall thickness (RWT). Logistic regression model was applied to determine the odds ratio (*OR*) and 95% confidence intervals (*CI*) of the risk factors of left ventricular hypertrophy.

**Results:**

The prevalence of LVH was 20.2% in untreated hypertensive patients, much higher in women (30.8%) than in men (14.2%) (P < 0.01). The prevalence of LV geometrical patterns was 34.9%, 11.1%, 9.1% for concentric remodeling, concentric and eccentric hypertrophy,respectively. After adjustment by using Logistic regression model, the risk factors for LVH and abnormal LV geometry were age, female, systolic blood pressure, and body mass index. And low high density lipoprotein maybe a positive factor.

**Conclusions:**

The prevalence of LVH and abnormal LV geometric patterns was higher in women than in men and increased with age. It is crucial to improve the awareness rate of hypertension and control the risk factors of CV complications in untreated hypertensive population.

## Background

In hypertensive patients, an adaptive myocardial response to increased cardiac afterload results in left ventricular hypertrophy (LVH) [1]. Hypertensive LVH is a powerful independent predictor for sudden cardiac death [2], ventricular arrhythmias [3], myocardial ischemia [4], coronary heart disease [5], heart failure [6], as well as ischemic stroke [7].

Echocardiography is more sensitive and specific than electrocardiography in the detection of LVH [8]. Classification of patients based on whether left ventricular mass index (LVMI) and relative wall thickness (RWT) are normal or abnormal yields four left ventricular (LV) geometric patterns: normal, concentric remodeling, concentric hypertrophy and eccentric hypertrophy [9]. Previous studies have reported that echocardiographically determined LV geometry can independently predict major cardiovascular events [10], and the worst is concentric hypertrophy, followed by eccentric hypertrophy, concentric remodeling and normal geometry [11]. In addition, LV geometric pattern is closely related to stroke risk [12].

The various prevalence of LVH and abnormal LV geometry have been reported in different populations [13, 14]. So far, only a few reports are available on the prevalence of LV geometric patterns in a large Chinese untreated hypertensive population. Therefore, we conducted a cross-sectional study to survey the prevalence of LVH and LV geometric patterns in untreated hypertension population in northern China.

## Methods

### Study population

This community-based cross-sectional study was conducted in the Rizhao City and Hong Xing Long County, in the northern region of China from 2009 to 2010. A multistage cluster sampling method was used. A total of 9,286 subjects (5167 men and 4119 women) completed the survey, yielding a response rate of 97.48%. Among them, 2984 hypertensive patients were identified and thoroughly examined. Hypertension was defined as diastolic blood pressure (DBP) of ≥90 mmHg, and/or systolic blood pressure (SBP) of ≥140 mmHg, physician diagnosis, or current medication for hypertension (as defined by WHO 1999). Untreated hypertension was defined as never receiving any antihypertensive treatment befor the study.

Patients were excluded if they had hypertrophic cardiomyopathy, ischemic heart disease, congenital heart disease, or other organic heart disease including valvular disease. Patients with secondary hypertension, either suspected or established, were excluded as well.

The study was governed under the most recent (2007–2008) version of the World Medical Association’s Declaration of Helsinki. The study protocol was reviewed and approved by the ethical committees of the Fuwai Hospital and local hospitals. Participation is voluntary; informed consent was obtained from each participant. All investigators were trained at the Cardiovascular Institute, Chinese Academy of Medical Sciences (Beijing, China) and qualified for the clinical investigation.

### Data collection

We identified eligible individuals according to their age and documents of residence and invited them to a community clinic by telephone. Each participant was interviewed and completed a standardized questionnaire that included a range of demographic factors, medical history, history of medications, and lifestyle.

### Physical examination

Anthropometric measurements of subjects who wore light clothing and were in bare feet were conducted by experienced research staff. Height was measured once to the nearest 0.1 cm, and weight was measured in the upright position to the nearest 0.1 kg.

BP was measured by trained professionals with a standardized mercury sphygmomanometer, and one of three cuff sizes (regular adult, large, or small) was chosen on the basis of the circumference of the participant’s right arm. All participants were advised to avoid alcohol, cigarette smoking, coffee/tea, and exercise for at least 30 minutes before their BP measurement. Three BP readings were recorded at least 1 minute apart in the sitting position after at least 5-minute rest and averaged for further analysis.

### Echocardiography

Transthoracic echocardiography was performed according to standard protocol [15] with M-mode, 2-dimensional (2D), and color Doppler recordings from the parasternal long-axis and short-axis windows, as well as 2D and color Doppler evaluations from the apical window to yield 2-, 3-, and 4-chamber images with an HP 5500 (Phillips Medical System, Boston, Massachusetts, USA). The transducer frequency was 2.5 to 3.5 MHz. Optigo echocardiographic recorders (Agilent, Boston, Massachusetts, USA) were used occasionally to screen subjects who could not reach the local study center. The echocardiographic examination was supervised by 2 physician-echocardiographers with at least 2 years of experience. Before the study, they were trained in the echocardiographic protocol at the Cardiovascular Institute, Chinese Academy of Medical Sciences.

### Calculation of derived variables

Left ventricular mass (LVM) was calculated using the equation:

LVM = 0.8 × 1.04 × [(IVSd + LVIDD + PWTd)^3^–LVIDD^3^] + 0.6, which yields values closely related (*R* = 0.90) to necropsy LV weight [16], where IVSd is septal wall thickness at end diastole, PWTd is posterior wall thickness at end diastole , and LVIDD is left ventricular end-diastolic diameter.

LVM was divided by height^2.7^ and body surface area (BSA) to obtain left ventricular mass index (LVMI_h2.7_ and LVMI_BSA_). BSA was calculated by using the Du Bois formula [17]: 0.0 071 843 × (weight (kg))^0.4253^ × (height (cm))^0.725^.

LV hypertrophy was diagnosed by using the criteria of the LVMI_h2.7_ more than 49.2 g/m^2.7^ and 46.7 g/m^2.7^ for males and females, respectively [18]. Relative wall thickness (RWT) [19] was calculated by 2 × PWTd/LVIDD.

The LV geometry was classified into four patterns based on LVMI and RWT [20] values:

1. Normal geometry: LVMI was normal and RWT was < 0.43;
2. Concentric hypertrophy: LVMI was increased and RWT was ≥ 0.43;
3. Eccentric hypertrophy: LVMI was increased and RWT was < 0.43;
4. Concentric remodeling: LVMI was normal and RWT was ≥ 0.43.

### Statistical analysis

Data are reported as mean ± standard deviation (SD) for continuous variables and as frequency for categorical variables. Differences in continuous variables between two groups were compared with a Student t-test and differences in categorical variables were measured with a chi-square test. Differences between multiple groups were performed by analysis of variance (ANOVA). Logistic regression was used to calculate odds ratios (ORs) and their 95% confidence intervals (CIs). Potential confounders were adjusted. A 2-tailed value of P < 0.05 was considered significant. Analyses were performed with SPSS 11.0 (SPSS Inc, Chicago, USA) for Windows (Microsoft Corp, Redmond, USA). The authors had full access to the data and take full responsibility for its integrity.

## Results

### Clinical and echocardiographic characteristics of untreated hypertensive population

A total of 1641 untreated hypertensive patients (1044 males and 597 females) with integrated clinical and echocardiographic data enrolled in the present study (Table 1 and Table 2).

**Table 1:** **Clinical characteristics of 1641 untreated hypertensive patients**

Variables	Whole group (n = 1641)	Male (n = 1044)	Female (n = 597)	p value
**Age (years)**	50.4 ± 12.18	47.8 ± 12.26	55.1 ± 10.53	<0.001
**Height (cm)**	165.6 ± 8.76	170.3 ± 6.47	157.5 ± 5.90	<0.001
**Weight (kg)**	73.5 ± 12.97	78.7 ± 11.76	65.6 ± 9.75	<0.001
**BMI (kg/m** ^**2**^ **)**	26.7 ± 3.38	27.1 ± 3.28	26.0 ± 3.50	<0.001
**BSA (m** ^**2**^ **)**	1.8 ± 0.19	1.9 ± 0.16	1.7 ± 0.13	<0.001
**SBP (mmHg)**	139.5 ± 15.29	138.8 ± 14.69	140.7 ± 16.23	0.013
**DBP (mmHg)**	92.0 ± 9.34	93.7 ± 8.83	88.9 ± 9.43	<0.001
**PP(mmHg)**	47.5 ± 15.52	45.1 ± 14.19	51.8 ± 16.79	<0.001
**MAP (mmHg)**	107.8 ± 9.09	108.7 ± 8.90	106.2 ± 9.19	<0.001
**Plasma glucose (mmol/L)**	5.5 ± 1.86	5.4 ± 1.98	5.7 ± 1.59	<0.001
**Cholesterol (mmol/L)**	5.4 ± 1.05	5.5 ± 1.05	5.3 ± 1.03	0.017
**Triglyceride (mmol/L)**	2.0 ± 1.54	2.2 ± 1.78	1.7 ± 0.97	<0.001
**High density lipoprotein (mmol/L)**	1.5 ± 0.36	1.4 ± 0.37	1.5 ± 0.35	<0.001
**Low density lipoprotein (mmol/L)**	3.0 ± 1.02	3.0 ± 1.08	3.0 ± 0.91	0.727
**Diabetes (%)**	94(5.7)	46(4.4)	48(8.1)	0.003
**Obesity (%)**	252(15.4)	176(16.9)	76(12.7)	0.027

BMI = Body Mass Index, BSA = Body Surface Area, SBP = Systolic Blood Pressure, DBP = Diastolic Blood, PP = Pulse Pressure, MAP = Mean Arterial Blood Pressure.

**Table 2:** **Echocardiographic characteristics of 1641 untreated hypertensive patients**

Variables	Whole group (n = 1641)	Male (n = 1044)	Female (n = 597)	p value
**IVSd (mm)**	10.6 ± 2.14	10.5 ± 2.20	10.7 ± 2.03	0.053
**PWTd (mm)**	9.4 ± 1.54	9.5 ± 1.46	9.1 ± 1.64	<0.001
**LVIDD (mm)**	44.4 ± 5.25	45.3 ± 4.97	42.9 ± 5.37	<0.001
**LV mass(g)**	152.5 ± 46.43	158.1 ± 48.04	142.5 ± 41.69	<0.001
**LVMI-BSA(g/m** ^**2**^ **)**	84.5 ± 24.70	83.5 ± 25.13	86.2 ± 23.84	0.027
**LVMI-height** ^**2.7**^ **(g/m** ^**2.7**^ **)**	39.4 ± 12.60	37.8 ± 12.24	42.0 ± 12.79	<0.001
**RWT(cm)**	0.43 ± 0.092	0.43 ± 0.085	0.44 ± 0.102	0.117

IVSd: end-diastolic interventricular septal thickness; PWTd: end-diastolic posterior wall thickness; LVIDD: end-diastolic LV internal dimension; LVMI_BSA_: left ventricular mass index divided by body mass index; LVMI_h2.7_: left ventricular mass index divided by height^2.7^; RWT: relative wall thickness.

LVIDD as well as PWTd were larger in men than in women, so did LV mass (158.1 ± 48.04 g vs. 142.5 ± 41.69 g, P <0.001). The trend was opposite after indexed by height^2.7^ (37.8 ± 12.24 vs. 42.0 ± 12.79, P <0.001), and by BSA (83.5 ± 25.13 vs. 86.2 ± 23.84, P =0.027). Moreover, RWT was higher in women, but this difference did not attain statistical significance.

### Prevalence of LVH in untreated hypertensive population

**Figure 1:**
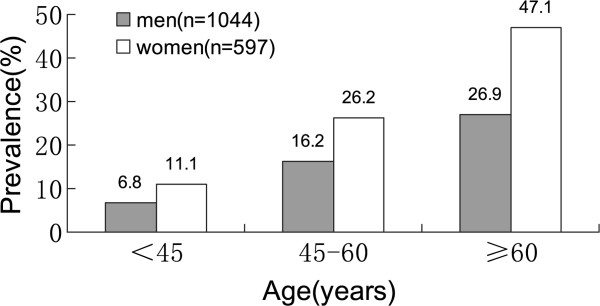
**Prevalence of left ventricular hypertrophy (LVH) in different age and sex groups.** The prevalence was much higher in women than in men in the age groups of 45–60 and ≥60 (p < 0.01), and increasing with ageing.

Of 1641 untreated hypertensive patients, 20.2% (n = 332) was found to be LVH, 14.2% in men and 30.8% in women respectively. Sex-specific prevalence of LVH increased with ageing (Figure 1).

### The distribution of LV geometric patterns

The total distribution of LV geometric patterns was concentric hypertrophy (11.1%), eccentric hypertrophy (9.1%), concentric remodeling (34.9%) and normal geometric (44.9%). Concentric remodeling was the most common abnormal LV geometric pattern in men (35.9%), also, in women (33.0%). The LV geometric abnormality increased steadily with ageing (from 43.8% to 71.0%) (Table 3).

**Table 3:** **The distribution of LV geometry patterns**

Subgroup	Normal geometry [***n***(%)]	Concentric remodeling [***n***(%)]	Concentric hypertrophy [***n***(%)]	Eccentric hypertrophy [***n***(%)]	Abnormal geometry [***n***(%)]
**Sex**					
**Male**	521(49.9)	375(35.9)	74(7.1)	74(7.1)	523(50.1)
**Female**	216(36.2)	197(33.0)	108(18.1)	76(12.7)	381(63.8)
**Age group (years)**					
**<45**	305(56.2)	197(36.3)	20(3.7)	21(3.9)	238(43.8)
**45-60**	317(45.2)	241(34.4)	75(10.7)	68(9.7)	384(54.8)
**≥60**	115(29.0)	134(33.8)	87(21.9)	61(15.4)	282(71.0)
**Total**	737(44.9)	572(34.9)	182(11.1)	150(9.1)	904(55.1)

### The risk factors of LVH in untreated hypertensive patients

After adjusted for age, sex, systolic blood pressure, diastolic blood pressure, body mass index, cholesterol, triglyceride, high density lipoprotein cholesterol, low density lipoprotein cholesterol, smoking history, drinking history, history of diabetes,the following risk factors were identified by using Logistic regression model for confounders: age, female, SBP, BMI (Table 4).

**Table 4:** **The risk factors of LVH**

Variables	Odds ratio	95% CI	P
**Age,year**	1.06	1.05-1.07	<0.001
**Sex (0 = male,1 = female)**	1.94	1.38-2.74	<0.001
**SBP,mmHg**	1.02	1.01-1.03	<0.001
**BMI, kg/m** ^**2**^	1.19	1.14-1.24	<0.001
**Cholesterol, mmol/L**	0.876	0.759-1.010	0.069
**HDL, mmol/L**	0.66	0.42-1.05	0.079
**Drinking history (0 = no,1 = yes)**	0.69	0.47-1.02	0.060

Odds ratio was relative to no LVH. Adjusted for age, sex, systolic blood pressure, diastolic blood pressure, body mass index, cholesterol, triglyceride, high density lipoprotein cholesterol, low density lipoprotein cholesterol, smoking history, drinking history, history of diabetes. *P* value for variables to enter or stay in the model was set at <0.10. CI, confidential interval.

### The risk factors of abnormal LV geometric patterns in untreated hypertensive patients

After adjusted for age, sex, systolic blood pressure, diastolic blood pressure, body mass index, cholesterol, triglyceride, high density lipoprotein cholesterol, low density lipoprotein cholesterol, smoking history, drinking history, history of diabetes by using Logistic regression model for confounders, the risk factor of concentric remodeling was only age. The risk factors of concentric hypertrophy were age, female, SBP, BMI. The risk factors of eccentric hypertrophy were age, female, SBP, BMI, and high density lipoprotein and drinking history was found as protective factors for eccentric hypertrophy (Table 5).

**Table 5:** **The risk factors of abnormal left ventricular geometric patterns**

Variables	Concentric remodeling			Concentric hypertrophy			Eccentric hypertrophy		
	Odds ratio	95% CI	P	Odds ratio	95% CI	P	Odds ratio	95% CI	P
**Age,year**	1.02	1.01-1.03	<0.001	1.07	1.05-1.09	<0.001	1.06	1.04-1.08	<0.001
**Sex**									
**(0 = male,1 = female)**	1.20	0.93-1.55	0.158	2.51	1.70-3.71	<0.001	1.76	1.07-2.87	0.025
**SBP,mmHg**	1.00	0.99-1.01	0.615	1.03	1.02-1.04	<0.001	1.02	1.00-1.03	0.020
**BMI, kg/m** ^**2**^	0.99	0.95-1.03	0.567	1.17	1.11-1.24	<0.001	1.21	1.14-1.29	<0.001
**HDL, mmol/L**	0.92	0.64-1.31	0.651	0.95	0.54-1.69	0.865	0.37	0.18-0.73	0.004
**Drinking history (0 = no,1 = yes)**	1.00	0.75-1.33	0.974	0.76	0.44-1.31	0.321	0.53	0.31-0.94	0.028

Odds ratio was relative to normal geometry pattern. Adjusted for age, sex, systolic blood pressure, diastolic blood pressure, body mass index, cholesterol, triglyceride, high density lipoprotein, low density lipoprotein, smoking history, drinking history, history of diabetes. *P* value for variables to enter or stay in the model was set at <0.10. CI, confidential interval.

## Discussion

In the present study, the prevalence of LVH was 20.2% in the untreated hypertensive patients, while the prevalence of echocardiographic LVH was 42.8% among community-based hypertensive population previously reported by our group [21]. In other studies, the prevalence of LVH in untreated hypertensive cohorts was quite different, from 19% to 48% [18, 22, 23]. The distribution of abnormal LV geometric patterns was 34.9%, 11.1% and 9.1% for concentric remodeling, concentric hypertrophy and eccentric hypertrophy in this study, respectively, while our group found the distribution was shown to be 24.7%, 22.6%, 20.2% respectively in hypertensive patients [21]. Concentric remodeling was the most prevalent type of abnormal LV geometry in both sexes. Concentric hypertrophy, a LV geometric pattern related to a worse CV prognosis [24, 25], was more prevalent than eccentric hypertrophy , while lots of studies had come to the opposite conclusion [26–30]. The variation might result from the differences in age, gender, geographical region, diagnostic criteria, and risk factors.

It is worth noting that female was strongly associated with the prevalence of LVH and abnormal LV geometry in this study. This is in line with the majority of reports [31, 32], as well as our previous study [21].

Ageing was also related with increased prevalence of LVH and abnormal LV geometry in untreated patients. These data were in line with our previous study [21]. De Simone *et al.* [33]. Have also reported that, in both normotensive and hypertensive patients, left ventricle mass steadily increases with age. The Bogalusa Heart Study indicates that age-related increase in left ventricular mass could be partially interpreted by changes in body size and in BP [34]. Due to the aging of general population in China, it demonstrated that the incidence of LVH would increase. Also, both SBP and BMI were associated with LVH after adjusted for other risk factors, consistented with the result of published studies [32, 35].

Furthermore, a further aspect of our study was that maybe low high density lipoprotein was associated with higher prevalence of eccentric hypertrophy after adjusted for other risk factors.

The advantages of the study is that, this is a good community-based survey design, the routine ascertainment of LVH, and the definition of risk factors, survey data and echocardiography, which are similar to other well-known community-based studies.

In addition, another aspect of the present study deserves to be briefly discussed. A remarkable portion of untreated hypertensive patients enrolled in this study have had the manifestation of cardiac damage such as LVH, this suggests that primary prevention of cardiovascular disease is extremely crucial and claims for raising awareness of hypertensive status and controling risk factors for cardiovascular disease.

Several limitations applied to our study. First, our findings are not fully applicable to Han Chinese, as our samples comprised untreated hypertensive residents in the northern region of China. Second, the cross-sectional nature of this study limits the prediction of abnormal LV geometric patterns on cardiovascular and cerebrovascular events, which should be confirmed with a prospective study.

## Conclusions

Our study indicates that, in untreated hypertensive patients, LVH and abnormal LV geometric patterns is a predilection for female and the prevalence of LVH and abnormal LV geometric patterns increased with age.

Age, female, SBP, BMI are the major risk factors for LVH and abnormal LV geometric patterns. Also, low high density lipoprotein maybe has a positive effect on LVH.

Even though improved diagnosis and treatment of hypertension recent years, primary prevention of cardiovascular (CV) disease is extremely crucial. To effectively reducing of CV complications, it is of utmost importance that not only weight and BP control but also improving the awareness rate of hypertension in China and the risk factors of CV complications.

## Abbreviations

LV geometry: Left ventricular geometry
LVH: Left ventricular hypertrophy
LVM: Left ventricular mass
LVMI: Left ventricular mass index
IVSd: Septal wall thickness at end diastole
PWTd: Posterior wall thickness at end diastole
LVIDD: Left ventricular end-diastolic diameter
RWT: Relative wall thickness
SBP: Systolic blood pressure
DBP: Diastolic blood pressure
BSA: Body surface area
CV: Cardiovascular.
